# Non-Invasive Data Acquisition and IoT Solution for Human Vital Signs Monitoring: Applications, Limitations and Future Prospects

**DOI:** 10.3390/s22176625

**Published:** 2022-09-01

**Authors:** Mahmoud Salem, Ahmed Elkaseer, Islam A. M. El-Maddah, Khaled Y. Youssef, Steffen G. Scholz, Hoda K. Mohamed

**Affiliations:** 1 Institute for Automation and Applied Informatics, Karlsruhe Institute of Technology, 76344 Eggenstein-Leopoldshafen, Germany; 2 Karlsruhe Nano Micro Facility, Karlsruhe Institute of Technology, 76344 Eggenstein-Leopoldshafen, Germany; 3 Faculty of Engineering, Port Said University, Port Said 42526, Egypt; 4 Faculty of Engineering, Ain Shams University, Cairo 11535, Egypt; 5 Faculty of Navigation Science and Space Technology, Beni-Suef University, Beni-Suef 2731070, Egypt; 6 College of Engineering, Swansea University, Swansea SA2 8PP, UK

**Keywords:** healthcare system, vital signs, vital data, non-invasive data acquisition, internet of things (IoT), machine-to-machine (M2M) communication, wireless sensor network (WSN), digital image processing, computer vision

## Abstract

The rapid development of technology has brought about a revolution in healthcare stimulating a wide range of smart and autonomous applications in homes, clinics, surgeries and hospitals. Smart healthcare opens the opportunity for a qualitative advance in the relations between healthcare providers and end-users for the provision of healthcare such as enabling doctors to diagnose remotely while optimizing the accuracy of the diagnosis and maximizing the benefits of treatment by enabling close patient monitoring. This paper presents a comprehensive review of non-invasive vital data acquisition and the Internet of Things in healthcare informatics and thus reports the challenges in healthcare informatics and suggests future work that would lead to solutions to address the open challenges in IoT and non-invasive vital data acquisition. In particular, the conducted review has revealed that there has been a daunting challenge in the development of multi-frequency vital IoT systems, and addressing this issue will help enable the vital IoT node to be reachable by the broker in multiple area ranges. Furthermore, the utilization of multi-camera systems has proven its high potential to increase the accuracy of vital data acquisition, but the implementation of such systems has not been fully developed with unfilled gaps to be bridged. Moreover, the application of deep learning to the real-time analysis of vital data on the node/edge side will enable optimal, instant offline decision making. Finally, the synergistic integration of reliable power management and energy harvesting systems into non-invasive data acquisition has been omitted so far, and the successful implementation of such systems will lead to a smart, robust, sustainable and self-powered healthcare system.

## 1. Introduction

Today, a huge technological revolution in healthcare systems is taking place due to advances in the Internet of Things (IoT) [1], bio-sensing [2], non-invasive/non-contact sensing [3,4], artificial intelligence (AI) [5], mobile applications [6] and cloud computing [7]. Existing healthcare systems are not adequately structured to serve the needs of a population with a life expectancy that is growing. Thus, human vital data acquisition/monitoring/sharing still faces a number of challenges that have not yet been addressed, and there is a huge demand for adaptable, customized and usable healthcare solutions [8,9,10,11,12,13,14].

Vital data acquisition is considered a key component in any healthcare system, particularly those that provide care for the elderly, children, and those who are permanent patients [15]. Here, vital data acquisition is defined as the sensing, acquiring, processing and interpretation of measured bio-signals in order to determine vital information (bio-information) which can be utilized to help in disease diagnosis [16,17]. Moreover, this information enables monitoring of those with ongoing and possible life-threatening conditions.

Acquisition of vital data is performed in three stages [18,19]:

1. The sensing stage converts physical features to electrical signals.
2. The processing stage converts the acquired signals into a form that is understandable for software algorithms/embedded platforms/computers.
3. The analysis stage extracts valuable features for taking decisions.

**Figure 1:**
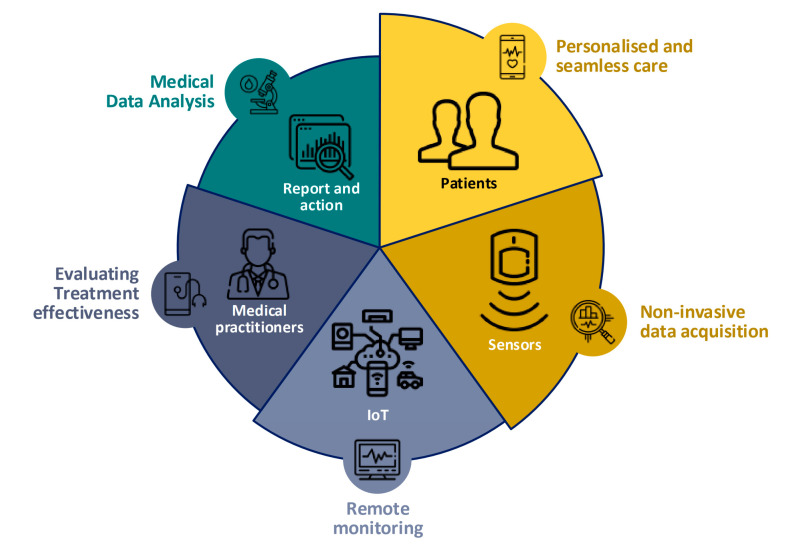
IoT is used in healthcare for varying purposes by different stakeholders (inspired from [27]).

The Internet of Things (IoT) can be defined as a communication solution which can be integrated/interfaced with different physical objects and software in order to enable information exchanges [13,20,21]. IoT technologies are proposed as one possible means of ameliorating the general shortage of resources for healthcare systems, at a time when life expectancy is increasing and medical costs are rising, by making the system more responsive and more cost-effective [21,22]. Indeed, IoT technology is considered to be an outstanding advance in the provision of healthcare services [23]. The development of sensors for vital data acquisition based on IoT integration enables data to be collected from patients effectively and promptly and analyzed for diagnoses and/or taking decisions [24,25,26]. A schematic of the development of effective healthcare based on the IoT is shown in Figure 1. The IoT provides full communication between sub-system/components/devices (things) in hospitals and real-time communication between patients and doctors to enable healthcare monitoring, diagnosis and supervision of treatment remotely. Figure 1 shows that the development of healthcare solutions via IoT algorithm can enable the acquisition of the full picture of the patient’s health status remotely. In addition, IoT algorithms facilitate real-time intercommunication between heterogeneous/different objects [22,27,28].

**Figure 2:**
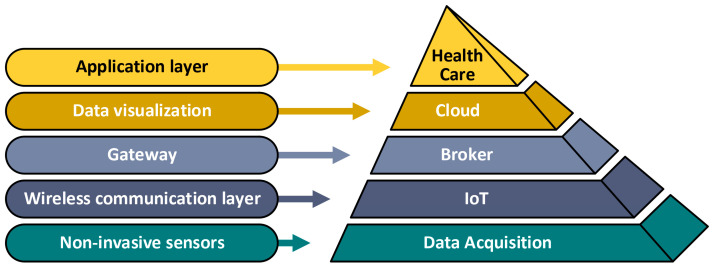
Hierarchy of healthcare solutions.

Vital data acquisition is the process of extracting human vital features/data for healthcare. IoT technologies enable intercommunication between healthcare subsystems (components) and/or things (physical world) and cloud computing (virtual world) [29,30]. The IoT is, at heart, a system that enables physical “things” (often sensors of one kind or another) to communicate and exchange information using suitable algorithms [31]. These exchanges of information enable sharing of knowledge between sub-system components in order to take the best decisions/actions based on accurate and up-to-date information. The IoT enables things to interact with cloud computing in order to process its information and receive decisions from the doctor via cloud’s computing solution [24,29,32]. How a combination of IoT and data acquisition enables modern and sustainable vital data acquisition is shown in the proposed hierarchy in Figure 2.

This paper describes the introduction of the IoT techniques into non-invasive vital data acquisition as part of health informatics and how such a development opens opportunities for healthcare systems as well as posing challenges. The changes from tele-healthcare to IoT, and from invasive to non-invasive data acquisition, are discussed. Recent research is reviewed to provide a knowledge base of data acquisition and use of the IoT in healthcare. The literature review of data acquisition concentrates on heart rate and body temperature. The design, development and use of the IoT in this area is covered and criticized. The existing challenges are highlighted and future implications in relevant directions, namely for enhanced IoT based non-invasive data acquisition systems, are suggested.

## 2. Internet of Things for Vital Data

With the rapid development in smart healthcare systems, it is foreseen that healthcare services will be transformed into hospitals-at-home in the coming years; see the recent study of the smart virtual hospital at home [33]. The technological revolution will enable full health monitoring for a patient’s lifetime, including vital signs monitoring, emergency situation alarms and medication management and telemedicine [34,35,36,37]. “Industry 4.0”, expects to transform the world of industry and the changes introduced will inevitably extend to other domains, including healthcare [29,38].

Elkaseer et al. (2018) reviewed practical approaches to the implementation of Industry 4.0 and defined the IoT as the capability to exchange information using smart sensor nodes (wireless sensor network-WSN) which are capable of adapting and enabling optimization of the desired application [29]. For healthcare solutions, Salem et al. (2019) defined IoT as a communication algorithm that enables the exchange of vital data to provide remote/internal vital data visualization and enable real-time vital data treatment [30], which agrees with [39]. The IoT could be a promising solution that overcomes the challenges associated with, for example, monitoring the vital data of disabled patients [40,41]. In one practical healthcare system, the IoT was developed using sensors as part of a WSN to detect and recognize relevant vital signs and irregularities [42,43]. In addition, IoT technologies such as radio-frequency identification (RFID), near-field communication (NFC), Zigbee, etc. facilitate communication between different healthcare sub-systems (things) such as visualization monitors, sensors, data acquisition units and servers [44,45,46]. Such communication sends the vital signs of patients to the doctors, remotely, wherever the patients’ locations. In the [47,48] studies of healthcare, IoT is proposed for specific goals such as detection of falls and/or seizures. In this way, IoT enables caregivers to receive a message/notification of the state of the patient and enable immediate corrective action to be taken.

**Figure 3:**
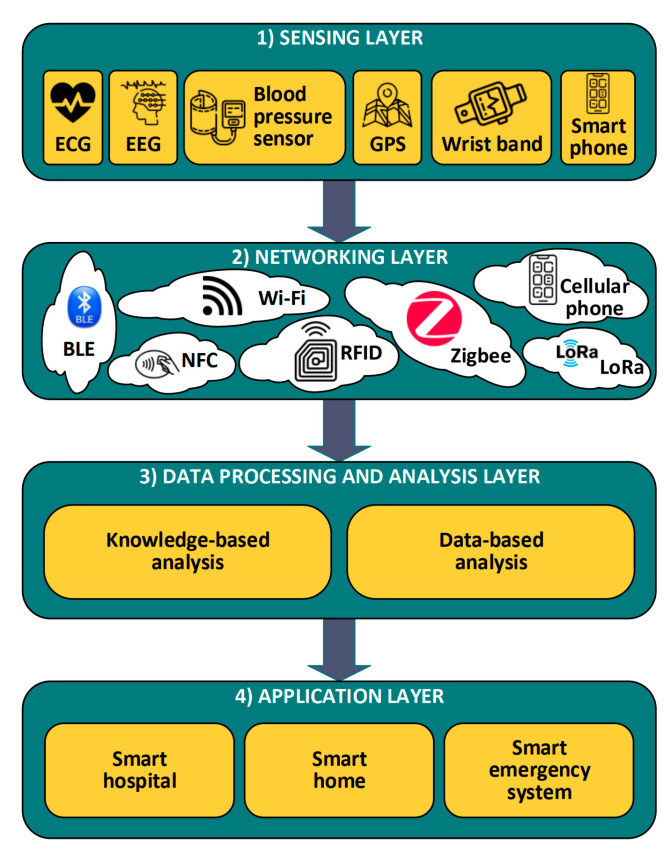
Layered architecture of IoT solution (modified from [60]).

Figure 3 shows a four-layered IoT architecture for human vital signs. Firstly, the sensing layer is devoted to observing the patients’ vital signs, such as blood pressure [49], heart rate [49], electrocardiography (ECG) signal [50], electroencephalogram (EEG) signal [51], etc. This layer collects the vital data from bio-sensors and processes it to find vital information/insights. However, in [52], the authors also introduced a means of locating the patient via a global positioning system (GPS) interfaced with an embedded system in the sensing layer. In the study by Cerlinca et al. (2010), RFID was integrated into the sensing layer to identify the patient [53]. In addition to healthcare/vital data acquisition systems, some home automation solutions integrate vital data acquisition [54], such as ambient assisted living (AAL), developed by Woznowski et al. (2015) [55].

The second layer provides fully secure/stable communication through the network/mesh and exchanges the information via communication technology such as Zigbee, Bluetooth Low Energy (BLE), IPv6 low-power wireless personal area network (6LowPAN), long-range radio technology (LoRa), etc. [56,57,58]. The third layer is the data processing layer, which provides effective processing of vital data in order to determine the patients’ status. The fourth layer, the application layer, can provide intelligent services and applications, such as those for disease diagnosis, behavior recognition and smart assistance [59,60].

A number of IoT technologies could be utilized for healthcare systems. These technologies are integrated with invasive/non-invasive vital data acquisition. Table 1 discusses the merits and demerits of different IoT techniques and technologies which can be utilized in e-healthcare systems and the table mentions a number of studies of IoT technologies in the vital data acquisition and e-healthcare domains.

### 2.1. Vital-IoT in Smart Homes

The monitoring of human vital data and human activity are very important both at home and in the working environment (including, schools, offices and manufacturing). Ambient assisted living (AAL) is promoted as a solution to the provision of health monitoring and care at home for elderly people who suffer from some form of disability [66]. AAL architecture has been developed by integrating sensor technologies, including body-worn sensors, cameras for activity recognition and environmental sensors to sense the local environmental conditions. Furthermore, AAL visualizes the sensed data and human activity on a user interface (UI). Then, the sensing data is treated and sent via a gateway to the cloud/doctors [67]. One of the AAL solutions is developed by Woznowski et al. (2015) [55] and was designed to enable patients to communicate with doctors and then, on advice from the doctors, where possible and appropriate, self-medicate.

**Figure 4:**
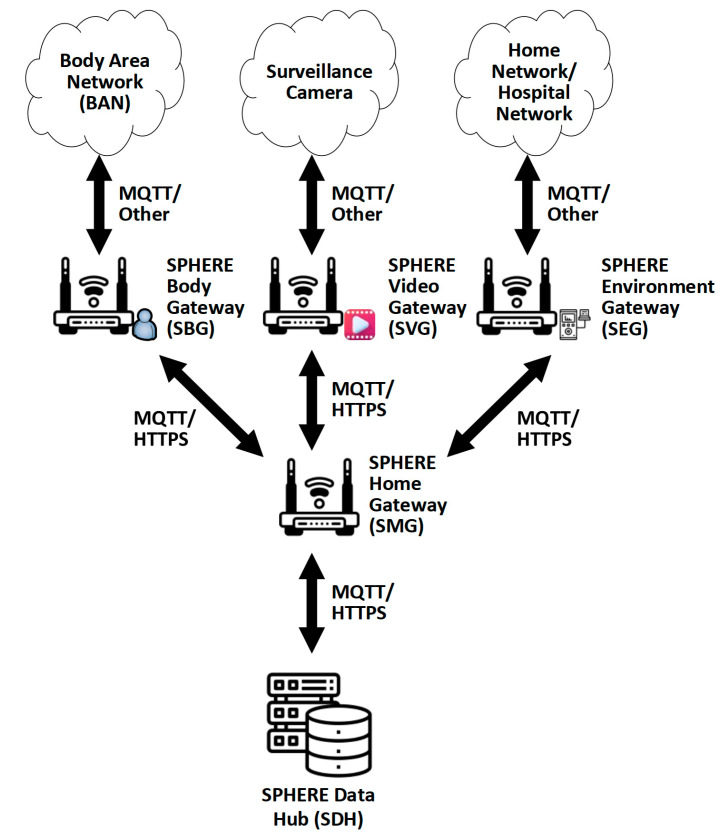
AAL system architecture (modified from [55]).

AAL-SPHERE is a multi-model that could be developed with advances in sensor and communications technologies to monitor the activities associated with daily living (ADL). In Figure 4, a message queue telemetry transport (MQTT) protocol enables communication between body, video, and environment sensor networks and with the broker (gateway). After that, the SPHERE data hub performs an analysis of the acquired data and, based on that analysis, the patient’s activities can be visualized using the cloud. Nevertheless, this solution was restricted to monitoring one patient and was not fully non-invasive. Some vital data was obtained using an invasive method which could increase the risk of infection. At this stage of development, the solution is restricted to home monitoring only. This architecture agrees with what is reviewed in [19] for remote sensing in COVID-19 situations.

Mainette et al. (2016) developed a solution, that can aggregate the bio-medical information from different sensors and send it to a remote server and from there send notifications/messages to caregivers or doctors [68]. In particular, the data is aggregated by the WoX sensors and analyzed during an integration middleware stage in order to determine vital information. The development was implemented on two levels. Firstly, the data was collected through different sensors via a communication technique based on BLE. Secondly, the data was analyzed to extract the necessary information and send it via the gateway using Wi-Fi technology.

In [68], the solution design was effective, but the prototype was physically large and no study was carried out on either the power consumed or on the effect on the wearer of wearing the device which can be self-powered wearable IoT node as mentioned in [69,70,71] or be consumed low power as developed in [72]. However, using an embedded Linux platform meant it was capable of undertaking many tasks due to it being high.

In order to obtain full reports on patients and send alerts to the caregivers, Coelho et al. (2015) developed a smart home architecture that was based on an iTech tool for sensing and a MySQL database. The developed solution was devoted to monitoring people with special needs [73]. The system used sensors and cameras to monitor human activity. These authors also developed an advanced algorithm to analyze the acquired data. This algorithm needed certain features to be included in the platform such as quick data transfer and high computational performance. The system combined two subsystems. Firstly the smart home module that used the iTech tool to collect data on human activities. Secondly, the cloud computing used a MySQL database that was part of Google Cloud to store and analyze the acquired data [73]. Nevertheless, the system was limited to sensing human behavior, i.e., the activities of people with special needs, rather than vital data.

Ray (2014) developed a framework known as the Home Health Hub Internet of Things (H3 IoT) for home health monitoring that sensed vital data [74]. The H3 IoT framework included five layers. Firstly, the physiological sensing layer included bio-sensors to monitor bio-functions such as heart rate, body temperature, blood glucose, EEG, respiratory rate, etc. Secondly, the local communication layer used a low range communication technique, i.e., radio frequency (RF), to transfer data among subsystems. Thirdly, the information processing layer performed the processing and analysis of vital data using computing platforms such as digital signal processing, embedded system platforms, or a field programmable gate array. These platforms interface with a high-level communication technique such as long-term evolution to transfer vital information for cloud computing [75,76,77]. Fourthly, the internet application layer that enabled the application to work through a mobile app or desktop app in order to manage solution parameters [78]. Fifth and last was the user application layer that interfaced with the healthcare providers and enabled them to perform diagnostic investigations and recommend possible solutions. This framework had certain advantages; the layers were clearly structured and its interface was user-friendly. However, the framework was difficult to upgrade and was not fully non-invasive when sensing vital data. Importantly it was restricted to home use and not applicable to more crowded environments. Furthermore, the authors did not apply their framework to a real system.

Jara et al. (2011) developed an IoT solution for diabetic therapy management in ambient assisted living (AAL) [79]. The solution aimed to provide the diabetic patient with guided advice to keep his/her blood glucose level within a safe range. The developed solution utilized the IoT to provide communication between a blood glucose sensor and the internet in order to monitor blood glucose ratios on which to base the advice given to the patient. The system was developed using various communication techniques such as 6LoWPAN and RFID.

### 2.2. Vital IoT in M-Health and E-Health

The IoT is not restricted only to smart home and AAL but has also enabled development of mobile healthcare (M-health) and electronic healthcare (E-health); in fact, the IoT opens the door to the universal provision of healthcare. The IoT means that solutions to health problems will not be restricted to patient–physician communication, but new, personally-tailored curative methods will be available to all patients. The IoT will enable full access and real-time analysis of medical data that could increase the quality of medical services and provision [80]. Additionally, IoT enables telesurgery and surgical telemonitoring [81]. The system developed in [80], termed smart space by the authors, allows doctor and patient to communicate whenever and wherever the patient wants it, whether at home, at school, at work. or at play. The design is as follows. First, there is the body area network that includes temperature sensors and other smart devices as worn by the patient. Secondly, the personal area network distributes environmental sensors in the smart space. Thirdly, the portable medical terminal is wearable by patients to detect an emergency state, which is agreed with [82]. The developed smart gateway allows an exchange of data throughout the whole sub-system components via the cloud server, which collects data from different resources and synchronizes them in the exchange processes.

For e-healthcare, the IoT enables medical devices to communicate/share data with each other via the internet in order to provide telehealth services. These enable direct communication between doctor and patient via the internet using smart devices that could be as advanced as robotically-assisted surgery. Additionally, it could provide full health monitoring for old and disabled people [83,84]. The telemedicine solution includes non-interactive consultation, where there is no real-time communication between patients and doctors, and instead communication is through the patient sending a report and receiving the doctor’s response, possibly as text messages. Interactive consultation might also use Skype, or Telegram, or a video link.

In [83], the authors reviewed a number of medical system applications using IoT. For example, Clinical Care, was developed using RFID technology to detect and define patients, to help with the monitoring of taking samples and doing tests. This solution monitors a patient’s location and facilities doctors/nurses to identify samples in an easy way.

Moreover, in another solution, which is called “Sickroom”, the solution provides vital data acquisition for patients in the sickroom. This vital data was acquired via sensors and sent to doctors and the care team via the internet.

M-health has applied tracking and monitoring of diseases over the long term, including diabetes and blood pressure [85,86]. In a recent study [87], a solution was provided for the monitoring of blood, temperature, oxygen saturation, heart rate and breathing via M-health concepts. The solution developed was based on four components. Firstly, physiological data acquisition. Secondly, an interface that included different types of communication techniques to enable communication from the system’s components. Thirdly, cloud computing that received data through the interface and gave its responses on a display that showed the vital data and responses.

Prouski et al. [88] developed a solution, where “smart glasses” measured eye pressure. The solution was developed using sensors which were in the frames of a pair of wearable glasses in order to detect eye pressure through the rate of blood flow in the human eyes. This solution sends notifications to the patient’s smart phone and makes an emergency contact in critical cases. The heart rate, blood pressure and eye presser have a correlation to each other [89,90,91].

**Figure 5:**
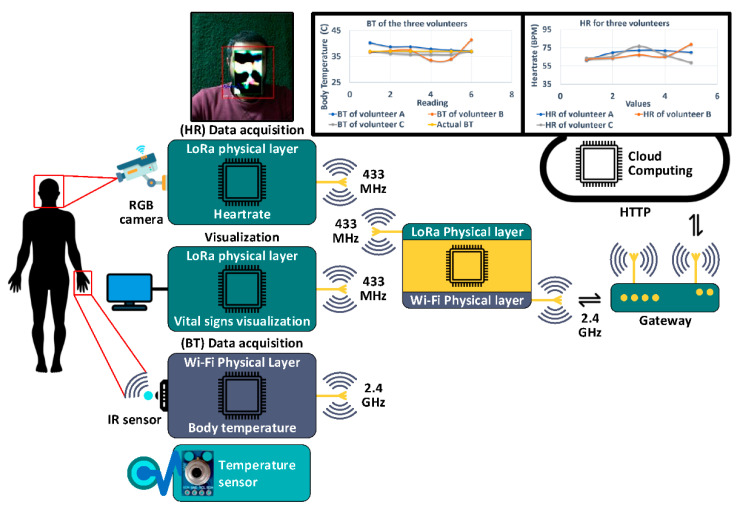
The architecture of IoT System for non-invasive vital data acquisition (modified from [30,39]).

Salem et al. [30,39] developed a dual-frequencies IoT network for non-invasive data accusation. A new design for a vital data acquisition using non-invasive algorithms was introduced (see Figure 5). The two vital signs monitored were heart rate, which was received via a non-invasive camera and body temperature that was extracted using infrared IR sensor. The proposed data acquisition algorithms were integrated into a new design of heterogeneous IoT architecture composed of four IoT nodes connected using two frequencies, 2.4 GHz and 433 MHz. This multi-frequency algorithm was introduced via embedded software for ESP32 and LoRa modules. Where the node for measuring heart rate propagated its data at 433 MHz, and the node for measuring body temperature propagated its data on 2.4 GHz. The broker node transmitted/received data over both 2.4 GHz and 433 MHz, as shown in Figure 5. The setup could be easily afforded by workplaces, hospitals, schools and domestic dwellings. Moreover, the solution is capable of being extended due to the capability of the broker node processing chip. 

### 2.3. Integration of Vital Data Acquisition into IoT Nodes

The integration of vital data acquisition into IoT nodes/devices could be carried out via different communication protocols which can be categorized as follows.

Both universal synchronous asynchronous receiver/transmitter (USART) and universal synchronous asynchronous receiver/transmitter (UART) work under full-duplex communication mode, which enables them to transmit and receive data at the same time [30,92], with the ability of the USART to adding synchronization feature. The synchronization feature enables the sender to produce a clock signal that can be received by the receiver. However, In the UART the clock signal is not required because the data stream is sent with defined baud rate ahead, and thus it is considered more cost-effective. In addition, both protocols (USART/UART) use a parity bit for error detection that increases the accuracy of sending data. The main limitation of these protocols is to restrict work with only one sensor because there is no communication facility with different sensors in the same communication bus. Additionally, these protocols have a limited baud rate for sending the data.

Inter-integrated circuit (I2C) is a serial communication protocol that facilitates connecting different sensors with a microcontroller. I2C requires two bidirectional active wires which are termed serial data line (SDA) and serial clock line (SCL) to exchanges the information between devices. I2C is a master to slave communication protocol, where each slave (sensor) has been defined with a unique address. In order to establish communication, the master device initially sends the target sensor address along with read/write (R/W) flag. The matching device/sensor (slave) will move into active mode, and other devices/sensors (slaves) will switch to offline state. Once the sensor is ready, communication starts between master (IoT node) and slave (sensor) [92,93]. This protocol provides good communication between onboard devices/sensors/nodes which are accessed infrequently. Additionally, its addressing mechanism ensures the correction of sending/receiving the information. Moreover, its cost and circuit complexity does not change with the increase in the number of sensors added. However, the I2C protocol has a limited sending speed [92,93].

Serial peripheral interface (SPI) is a serial communication protocol consists of a 4-wire protocol which are termed as master out slave in (MOSI), master in slave out (MISO), slave select (SS), and serial clock (SCLK). Similar to I2C protocol, SPI is also a master to slave communication protocol. In SPI, the master device first sets the clock at a particular frequency. Next, the SS line is used to select a slave by pulling the SS line low (zero pulse) where it is normally held high (one pulse). Then, the communication is established between the selected slave (sensor) and the master (IoT node) [94]. The SPI is faster than the serial communication protocol, and it supports multiple slave connectivity. Additionally, it is a low-cost communication protocol, and it is considered a universally accepted protocol. However, setting up a communication mesh for this protocol requires more wires than other communication protocols. Increasing the number of slave sensors could lead to circuit complexity [92].

## 3. Non-Invasive Vital Signs Acquisition

**Figure 6:**
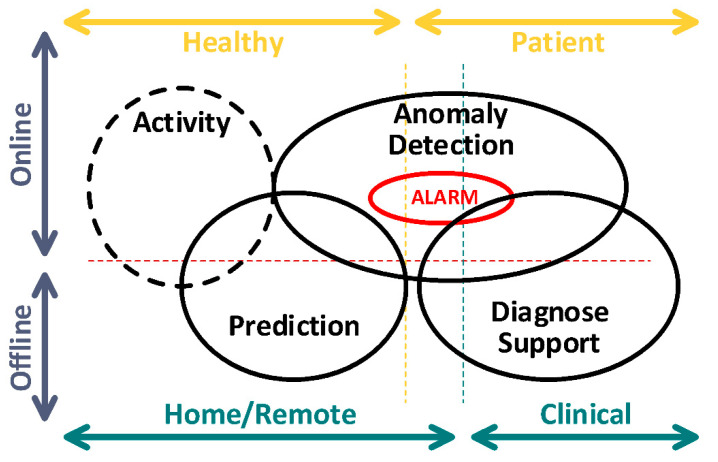
Scenario for vital sign acquisition: home, remote or clinical environment (modified from [96]).

Human vital data has multiple physiological dimensions which can be measured via invasive methods, i.e., sensors which touch the human body, or via non-invasive methods, such as cameras, magnetic resonance imaging (MRI), and computerized tomography (CT). These signals can be processed to clarify/identify features contained in the vital data to help doctors reach a correct diagnosis for a given patient [95]. In Figure 6, the vital data acquisition can be applied remotely or inside a hospital, either offline or online (real-time). Furthermore, it can be applied as a non-medical application, such as when monitoring fitness. The vital data acquired should contain sufficient useful information for anomaly detection, diagnosis and visualization of health records [96,97,98,99].

The acquisition of vital data is a technology that is ongoing and that should be applicable in all aspects of daily life: the home (especially when sleeping), at school, in clinics, in surgeries and hospitals and during leisure activities, particularly sporting activities.

The transformation of healthcare by taking it out of a clinical environment began in the late 1990s with development of the new concept, enabled by technological advances of providing vital data acquisition via wearable devices [99]. The objective was to introduce continuous health monitoring for patients. Improvements to the systems were introduced with multiple new technologies particularly information and communication technologies, bio-medical technologies and micro- and nano-material engineering [97,99]. Today, vital data acquisition can be achieved by a new generation of wearable heath devices in common use that can track heart and respiratory rates and monitor activity and sleep patterns, with real-time monitoring by healthcare professionals for patients and those with special needs. Such widespread and ongoing monitoring ensures better support for medical diagnosis and faster recovery from bodily injuries.

The solution for acquiring vital data through wearable devices should meet the following requirements [31,95,96,99,100,101,102]:

1. Be sustainable and consume low power [30,39,103];
2. Be reliable [104,105,106];
3. Be secure from cyber-attack [107,108,109];
4. Be comfortable for the user [110];
5. Be upgradable [20,29];
6. Be safe [111];
7. Provide a safe and sustainable environment [39,111].

### 3.1. Heart Rate Vital Sign

The heart rate is measured as a series of pulses collected by a vital data acquisition system which can contain sensors/cameras, interface circuits and a processing unit/microcontroller. The human heart rate can be sensed by electrocardiography or photoplethysmography (PPG) algorithms. Additionally, the heart rate sensor of the Apple watch uses infrared, visible-light LEDs and photodiodes to detect heart rate [112].

Poh et al. (2010) developed an algorithm to sense heart rate. The algorithm integrated the outputs of a photo transistor and LED which were setup and embedded inside the earphone. In particular, the algorithm calculated the heart beats according to the infrared reflection which changes because of the blood flow rate [113]. The infrared output voltage was converted to discrete values (digital values) via a 12-bit analog–digital converter. A 0.8–4.0 Hz bandpass filter was used to pass heartbeats frequencies only and reject other frequencies. The values of the heart rate are visualized on the mobile phone and can be seen as beats per minute (BPM), which can be presented in graphical form for numerous human activities, such as standing and walking. This device required a wearable device, and the patients have to insert an earphone which could increase the chances of an infection which would make the device unusable. Additionally, the performance of the developed solution could change with change of use of the earphone, i.e., listening to music of different types. Based on the same theory of [113], a recent study [114] developed a solution to measure the blood pressure of patients.

Mohammed et al. (2014) proposed data acquisition of heartbeats, sensed via electrocardiography and processed using a IOIO-OTG. The IOIO was connected to the mobile phone to upload the sensed data to the cloud. This operation was performed via developed mobile software. The cloud software performs pattern matching/analysis for the uploaded data in order to recognize dangerous health situations [115,116]. The solution shows the ECG signal in real-time on the mobile phone as well as sending it to the cloud. However, the solution was restricted to use with the mobile phone, which makes the developed approach unusable because the user has to connect the IOIO board to the mobile phone. Again, the authors did not mention the way to sense ECG signal in a non-invasive way. For diagnosing arrhythmias, the work was not restricted to utilizing mobile phones, and a non-contact, non-invasive monitoring system to measure and estimate the heart and breathing rate of humans using a frequency-modulated continuous wave was developed by [117]. Additionally, the results of Mohammed et al. (2014) agrees with the study of [118,119,120], which utilized radar to monitor vital signs such as the heart rate and breathing rate.

In addition to ECG and PPG, there are other methods to sense heart rate, such as the ballistocardiogram (BCG), which senses the hemodynamic forces imparted by the pulsating flow of blood around the body to measure hemodynamic and heart functions [121,122]. A set-up was developed by Giovangrandi et al. (2012) to study and compare ECG and BCG measurements. The study confirmed that BCG was capable of sensing heart rate, but not as distinctly as ECG. However, the setup was restricted to be invasive in the ECG and BCG sensing.

In addition to ECG and BCG, an accelerometer has been used as a sensor to sense the heart rate. Aarts et al. (2017) developed vital data acquisition using an accelerometer, and the data was sent to a computer and suitable software visualized it [123]. For a healthy subject, the authors found accelerometer-based pulse detection of heart beats had excellent sensitivity. Additionally, the authors found that accelerometer performance was not influenced by changes in position, and placement of the accelerometer was easy in the given conditions. However, the solution is unusable outside of a hospital due to the complexity of the setup such as the need for an accelerometer of sufficient sensitivity and the necessary pre-amplifiers, though most people now have a personal computer (PC). For the placing of the sensor, the patients had to attach the sensor to his/her neck which might not be easy at home.

**Figure 7:**
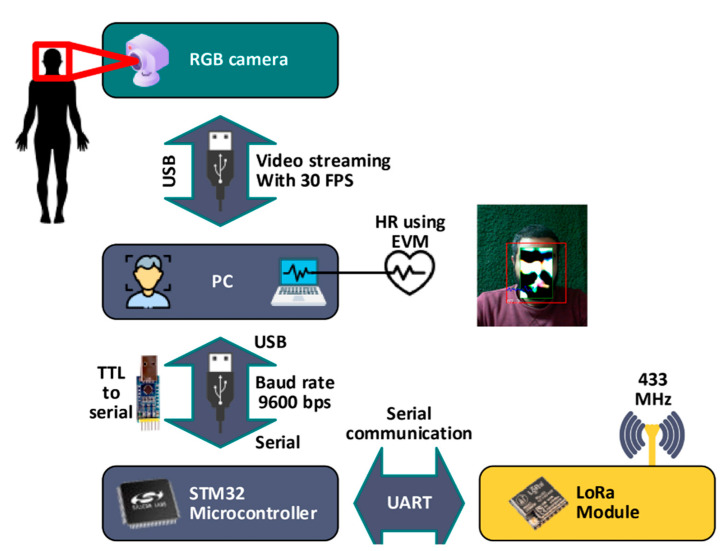
IoT nodes for estimating the heart rate using EVM (inspired from [30,39]).

In 2012, Wu et al. developed a new paradigm for non-invasive data acquisition. The authors developed an image processing algorithm for motion magnification [124]. The algorithm was developed using MATLAB [125]. The algorithm was run on 32 GB RAM and an Intel Core i7 microprocessor which facilitated a magnification algorithm that considerably enhanced, for example, the vital changes in human skin [124]. The colors changes refer to the changes in skin color brought about by blood flow and revealed the subject’s heartbeats. The algorithm was termed Eulerian video magnification (EVM) with a claimed accuracy comparable to hospital in-house measurements. The algorithm was extended to cover heart rate and respiratory rate in study [126]. Additionally, Wu et al. results is agreed with [127] in there developing of heart rate variability (HRV) as a risk assessment solution for diagnosing illnesses, and the results agreed with [128] for their developing of HR and eye-blinking monitoring using the vision system. Additionally, the methodology of extract (HR) from video is agreed with [129] which was utilized 3D central difference convolution with attention mechanism. The algorithm developed by Wu et al. combined both temporal and spatial filtering of the input from a video camera. At its simplest, to discern the effects of blood flow thorough, for example, the facial veins, a temporal band pass filter corresponding to the pulse rate (0.4 Hz to 3.0 Hz) might be used. A similar process is applied to the pixels. The solution used amplification factors of as much as 500, so effective noise filtering was essential for good results. The color of the face shown in Figure 7 can be seen to pulse between pallid and very flushed at a rate corresponding to the pulse rate. While very accurate, the algorithm requires a high level of processing resources, which is expensive. At the time, it was not capable of being integrated with the IoT.

Recent studies in [30,39] extended the work of [124] to be integrated with Internet of Things and to be run on low processing power platform, as shown in Figure 7.

In addition to implementing EVM on a high power machine, Chambino (2013) developed a non-invasive approach to extract the heart rate via a mobile phone based on the algorithm developed by Wu et al. Chambino developed the application using the Android Software Development Kit (SDK) and open computer vision library (OpenCV) to develop the phone as a low cost platform for the EVM application [130]. The value of the heart rate in BPM is printed in the mobile phone GUI. The application is capable of extracting the BPM in real-time, though the author did not say what methodology could be used to solve this issue in the mobile phone platform. Additionally, the author did not study the possible use of a mobile phone as an IoT node for M-health functions. The author of [131] extended this work with a nice GUI for desktop application based on Yolo4 for extract vital signs.

The sensing of heart rate is not restricted to using RGB video frames, it can also be sensed via thermal imaging. Garbey et al. (2007) developed a computer vision algorithm based on a thermal camera detecting cardiac pulses via thermal changes in the carotid artery by which it accurately determined the cardiac pulse rate [132]. However, a thermal camera is expensive and difficult to setup outside a hospital. The utilization of health monitoring for humans via computer vision can be extended to apply for animals as well [133]. These implementation of was extended to be implemented by 3D camera by [134]. It carried out good results but the setup was very expensive, and the author did not formalize the colorations between different vital signs affecting each other. Additionally, the utilization of convolutional neural network (CNN) and long short-term memory (LTSM) enhance the process of heart rate estimation using camera, as mentioned in [135].

### 3.2. Body Temperature Vital Signs

Body temperature (BT) is an important vital sign, and in this sub-section, the author covers recent solutions for BT determination. In [136,137,138], the body temperature was shown to be affected by the blood circulation and heart rate. In the developed solution in [138], the body temperature was estimated using a series of heart rate readings and a statistical model developed using a Kalman filter.

In a recent study [139], the authors developed a non-invasive vital data acquisition for human body temperate using microwave radiometry. The probe was a thin film attached to the skin to sense the body temperature and propagate the sensed value to a digital record. To make the data acquisition non-invasive the researchers made a number of important assumptions, the various layers of the body, skin, sub-cutaneous fat and muscle were uniform, and that the temperatures could be based on black-body power density curves.

**Figure 8:**
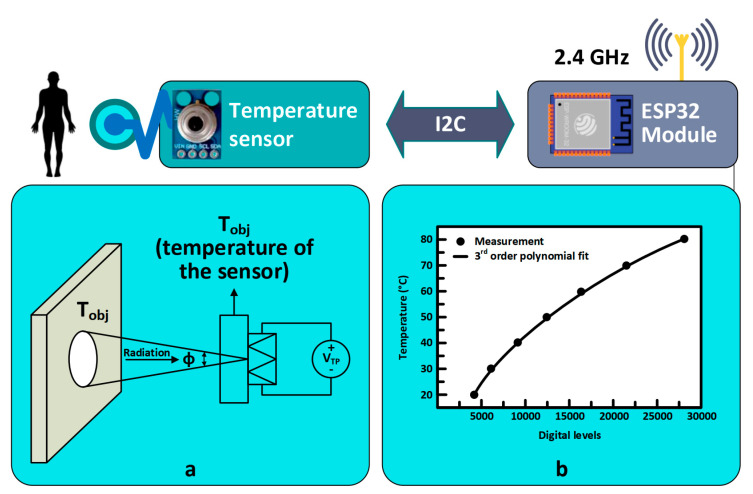
IoT nodes for estimating body temperature: (**a**) Conversion of the reflected radiation into voltage which refers to temperature values (**b**) Interpretation of digital signals into temperature values (inspired from [30,39]).

In [139,140,141], wearable devices as data acquisition systems for human body temperature measurement were developed. The solution was developed based on arrays of precise temperature sensors and provided real-time data of considerable accuracy (claimed as better than 0.1 °C). However, the solution was invasive and not user friendly. Furthermore, these solutions were not integrated into the IoT. In a recent example, in [63], the authors developed an epidermal temperature sensor for patients using a re-usable wireless temperature sensor to provide real-time vital data to the cloud. This approach calculated/estimated the temperate of the human body to an accuracy of between 0.6 and 2 °C, depending on where on the body the sensor was placed. The integration of RFID with the temperature sensor was designed in order to develop a remote batteryless RFID thermometer [63]. These measurements were performed with military personnel in a work situation. The author used a method which, while similar to a non-invasive technique, was not itself fully non-invasive. The implementation of non-invasive measurement of human body temperature retains many challenges. We note that in these studies the integration with a heterogeneous IoT network was not developed, as all the studies were restricted to the development of only one IoT node or homogenous IoT nodes. In [39], the authors developed heterogeneous IoT network for fully non-invasive data acquisition, which agrees with this approach of acquire the body temperature. The [39] results was agreed with a recent work of [142], which is graphed for further information in Figure 8. The limitation on this work is the health effect of RF on human body temperature because a recent studies found an effect for RF on human body temperature [143].

However, body temperature can be measured in an indirect way. Looney et al. developed a real-time method of measuring resting core temperature based on heart rate [144]. However, the solution was not easy to use.

**Figure 9:**
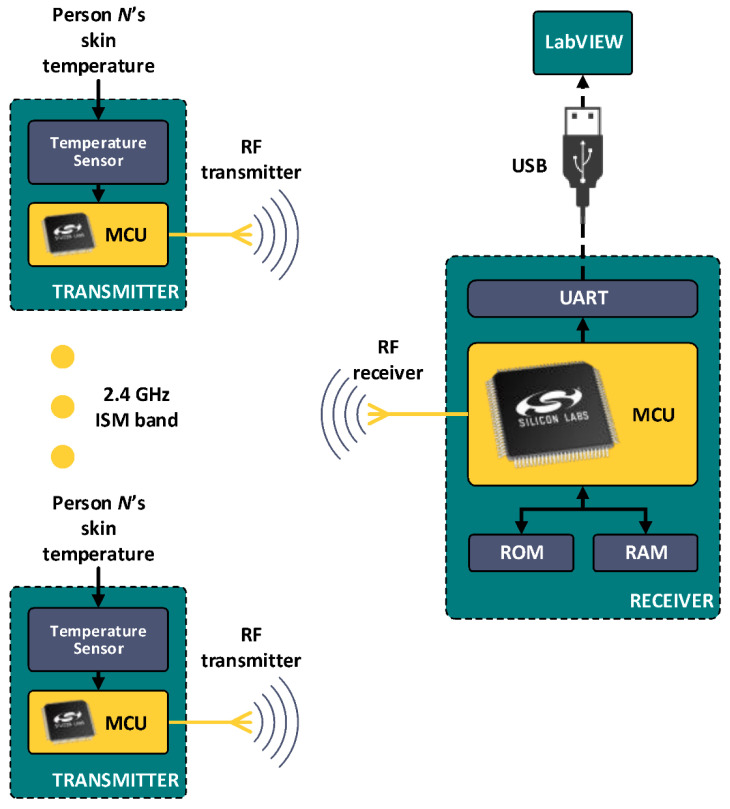
Block diagram of the Wei et al. wireless health monitoring system (modified from [145]).

In 2019, Wei et al. developed a cheap skin temperature sensing technique, accurate to ±0.3 °C but using an invasive method; for a diagram of the algorithm, see Figure 9. The temperature reading was sent via the IoT. The node was developed based on an ARM microcontroller to optimize the processing and power usage [145]. The structure of the sensing node contains temperature sensor, microcontroller unit (MCU) and RF transmitter. The diagram also shows the structure of the receiver which visualizes the received temperature using the software program LabView [146]. The printed circuit board (PCB) for the sensing node was made as small as possible and developed as a four-layer structure with surface mount devices located on both sides of the PCB.

**Figure 10:**
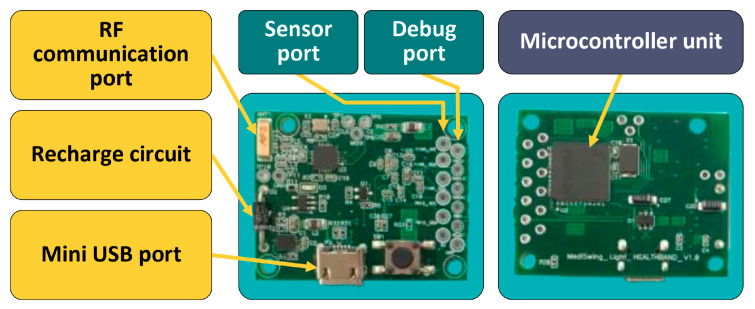
The developed PCB of wireless health monitoring (modified from [145]).

Figure 10 shows the PCB developed for the temperature sensing node. On the left of Figure 10 is the interface with the wireless module, on the right is the MCU attached in the PCB. The size of the node was small, with dimensions of 38 mm × 38 mm.

**Figure 11:**
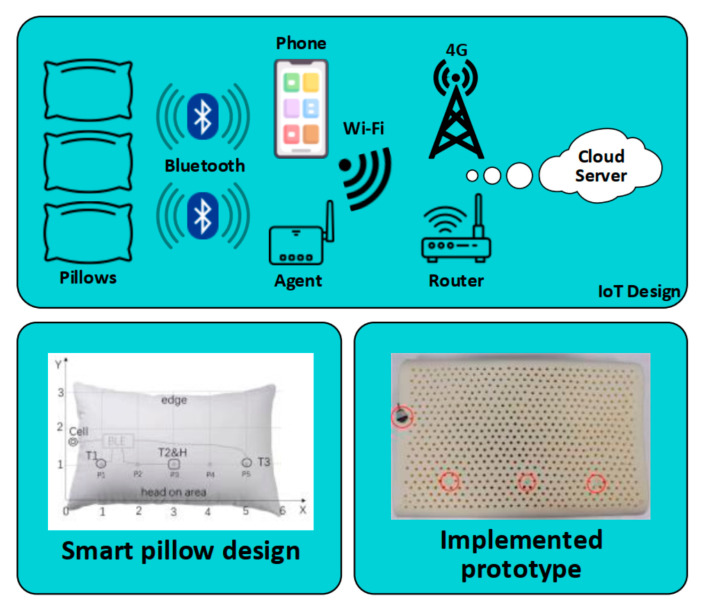
Smart pillow structure for temperature sensing (modified from [64]).

Li et al. (2018) developed a user-friendly body temperature measuring technique called the “smart pillow” (see Figure 11). The solution provides real-time body temperature sensing for via embedded sensors in the pillow. The sensors were connected to the broker node via BLE and a TMP112S Texas temperature sensor. The author utilized a mobile application that acted as a broker in order to access the gateway via Wi-Fi or 4G network. The mobile linked the vital data from different temperature sensors and calculates the vital features using a fuzzy logic control algorithm. The solution is very comfortable in use and consumes little power [64]. However, the technique was restricted to be used during sleeping. The authors did not mention the capabilities of integration with other IoT networks.

**Figure 12:**
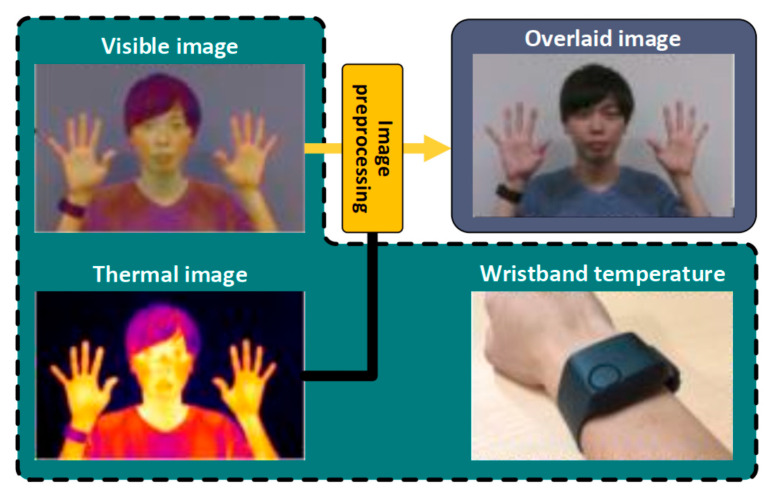
Human body temperature calculation using thermal camera and wristband temperature sensor (Modified from [148]).

The thermal camera has a high impact in enhancing non-invasive data acquisition [147]. Yoshikawa et al. developed a non-invasive thermal data acquisition system for the human body. The methodology was developed using a mobile phone with thermal camera attached. The image processing algorithm calculated the temperature of the person from the thermal camera frames and corrected the temperature using a wristband sensor (see Figure 12) [148]. The authors may have claimed the application was low cost but a mobile phone and thermal camera is relatively expensive. The system could not be considered fully non-invasive due to the use of a wristband sensor.

Deep learning has a high impact in healthcare system for estimating vital sign and in estimation of human activity [149,150,151,152,153,154]. In [155], an algorithm was developed using deep learning in order to sense the vital sign such as human body temperature using a thermal camera. The algorithm was developed based on the object detectors YOLOv4 and YOLOv4-Tiny, and the algorithm was trained using a dataset of 26 patients documented in an ICU.

### 3.3. Sensing Techniques for Vital Data Acquisition

There are several sensing techniques/technologies could be utilized for extracting the human vital signs, such as sensors, RGB cameras, 3D cameras, IR cameras, etc. Some commercial and fabricated/developed sensor techniques are discussed in Table 2. Additionally, embedded interfacing methodologies are mentioned with their features.

## 4. Review Summary, Perspective for the Future Work, and Open Challenges

### 4.1. Review Summary of IoT and Vital Data Aqcusition for e-Healthcare Systems

Table 3 discusses different recent studies of IoT for vital data acquisition and telemedicine which provide smart e-healthcare systems. The table discusses the sensing technologies and the IoT technologies. Additionally, the advantages and limitations of every study are reported.

### 4.2. Limitations, Open Challenges and Future Procpects in Non-Invasive Vital Data Acquisition

Looking at the previously reviewed literature, one can conclude that RGB cameras, radar, and radio frequency have been utilized to estimate heart rate (HR), body temperature (BT) and other vital signs. For the utilization of RGB cameras, the head rotation and hand position over the face, the illumination level of the environment, camera resolution and the number of frames negatively affect the accuracy of the algorithms such as the HR estimation algorithm [30,39,124,126,128,129,130,132,133]. For the future work, it is recommended to utilize multiple RGB cameras to obtain high accuracy with face orientation, and it is recommended to test many illumination levels with RGB color space to avoid the negative effect of high-low illumination. Additionally, the impact of emotions such as the psychological states of the patient can disrupt the estimation algorithm. So, it is recommended to develop a machine learning algorithm to analyze the HR video and study the effect of emotion on the estimated HR results to obtain more accurate results [161]. For a low number of frames per second (low temporary information), it is still an open challenge to perform Earlean video magnification for estimating vital data with low temporary information. Solving this challenge will optimize the algorithms for the low processing platform, so it will minimize the cost. For software development optimization, according to [30,128], the authors optimized the EVM algorithm to make the software of data acquisition able to run in low-spec machines. The optimization in software to make it able to be ported on embedded system kits is still an open challenge and its results will have a high positive impact in the market.

The utilization of DNN, such as CNN, for estimating heart rate has a good approach to perform HR estimation without complex analysis and feature extractors for the EVM problem. However, this may limit further development because DNN is a black box model [133].

For FIR imaging (thermal imaging), authors developed algorithms to estimate the body temperature [147,148], but there are some limitations that have to be addressed. One of these limitations is the estimation of the BT without reference point in order to increase the usability. Additionally, the study of the effect of room temperature on the thermal frame is still an open challenge and deep learning may help address it, but firstly an experimental dataset has to be collected in different conditions.

The chip design for data acquisition with its IoT module will affect a high impact in production. For example, a portable fabricated module which has its IoT layer, data acquisition layer and its self-powered system will be integrated as well. The fabrication of these three technologies in one chip as SoC will affect a positive impact in health informatics.

### 4.3. Limitations, Open Challenges and Future Procpects in IoT for Healthcare

From the reviewed literature, it is possible to emphasize that the enabling of IoT system to be more scalable to cover many areas and multiple nodes is limited. Salem et al. showed how to work with different frequencies as a step to be scalable, but there is no clue about how can it be scalable for millions of patients where every patient has 3–5 vital signs which have to be analyzed and sent to cloud computer in a short time. This is still an open challenge and needs appropriate addressing.

The second challenge is how to make the network of IoT have a mobility feature. Salem et al. [39] proposed a dual frequency approach, but the acceding of the gateway was only restricted by Wi-Fi frequency. The open question of how can the network cover the missing of accessing the broker remains. The system has to be flexible to switch to another backup frequency and check the facility to access the cloud through it [20] and design a backup for the loss connection with the could computer, but, unfortunately, there are limited studies to facility this approach on vital IoT systems.

The third challenge is the effect of wireless signals on the sensor reading if the sensor depends on the RF. There is a need to study the effect of different IoT frequencies on the RF-based sensors reading vital data.

The fourth challenge is the lack of standardization which mean there are no slandered SDN for healthcare, and if there is no standardization, the facility of tracking abnormal signal/data features will be difficult, and the system will be easily attached.

Power control is based on the design of data sending and the execution of SW, as developed and mentioned in [72].

### 4.4. Next Generation of e-Healthcare Systems with the Aid of Metaverse Technology

The explosive growth of digital technologies such as IoT, non-invasive vital data acquisition, artificial intelligence, virtual reality and augmented reality has led to the advent of a new level of e-healthcare systems based on metaverse technology [162]. In metaverse-based healthcare a non-invasive data acquisition system acquires the vital signs of patients and sends these signs to the doctor in emulated form using IoT. The doctor interfaces with this emulator using augmented reality and utilizes IoT to send the real-time actions/prescriptions to the patient without the need for in-person interactions. The integration of AI in metaverse applications provides decision-support systems which help doctors in diagnosis and treatment processes. This decision-support-based AI system uses the collected medical data which is collected during virtual medical investigation [163,164]. According to this working scenario, the metaverse-based healthcare helps monitor patients, analyze vital data, facilitate graded diagnosis, monitor treatment, prescribe personalized and precision medicine and follow up patient status remotely and seamlessly [165]. This approach can virtually interconnect doctors and patients around the clock and thus overcome the distance obstacle, especially for patents living in rural areas. In addition, the virtual training and education of junior doctors and medical students is another application where the metaverse can significantly contribute to by offering virtual training session similar to those in physical clinical practice, and thus better results can be obtained with less effort and almost zero risk [163].

The transition from research and development into industrial and commercial e-healthcare solutions plays an important role in enhancing medical services and reducing their costs. Specifically, IoT and non-invasive data acquisition are able to provide accurate, user-friendly, safe, remote and low-cost medical service. There is a need to build a business model for e-healthcare systems based on IoT and non-invasive data acquisition to move from research to industry. The following are the five questions to be answered to develop a proper business model [166], and the answers to these five questions are listed in Table 4.

## 5. Conclusions

This paper has reported recent advances in vital data acquisition and use of the IoT in healthcare informatics. A comprehensive review has shown that the non-invasive acquisition of vital data still faces many challenges before it can be practically implemented on a large scale. Non-invasive data acquisition is still limited both in terms of on-line, real-time sensing applications and in integration with the IoT. In addition, the use of the IoT in health informatics is still limited, and needs to be developed to cope with different transmission differences by using different frequencies, to use multiple bandwidths for transmission/receiving, and integration with non-invasive data acquisition.

What is required is a non-invasive means of acquiring vital data in real time, which is based on an accessible and easy-to use algorithms. Moreover, it has to be integrated with the IoT to enable real-time communication between patient and doctor. This could be achieved by using a suitable high-speed digital signal processing unit that can perform non-invasive data acquisition in real-time. This solution of the challenges will optimize the usage of vital data acquisition in real-time and minimize infection and accelerate reaction times. Moreover, it will provide a continuous report of a full picture of the patient’s condition.

A multi-frequency structure for IoT sensor nodes and an IoT broker can enable communication over long distances using different frequencies. The design of the broker is not likely to be simple and needs to be developed. Such a development will need well-designed software architecture. Such a development should introduce stable, secure and flexible communication between nodes in order to enable increasing of node numbers. Communication with different frequencies will enable data to be communicated over different distances via specific bandwidths.

The following points summarize the state of the art based on the reviewed literature.

(a) Computer vision algorithms can be utilized to extract the heart rate vital signs in real-time without requiring high processing power. On the other hand, the user is limited to using the computer vision application in a stable environment with not too bright, nor too dull, nor too noisy illumination in the captured frame because it increases the error of the heart rate estimation algorithm. Moreover, the subject has to place his/her face towards the camera without any orientation of the head, and care must be taken not to touch or obscure the face with a hand. Additionally, the algorithm for heart rate was also sensitive to the subject taking deep breaths or exhaling strongly. This suggests the computer vision solution may not be convenient for use in, for example, the intensive care units in hospitals but could be used generally in most internal environments.
(b) The body temperature can be sensed via IR without incurring any high costs of thermal cameras. The review shows that measuring the body temperature using a finger is not sufficiently accurate to be useful, and the sensing process has to target the middle of the forehead. The IR body temperature sensing can be affected by the environmental temperature if it is extra low/high, such as 15 °C or 35 °C, so it is recommended to use the IR sensing at “room temperature”, around 23 °C. The error of the developed algorithm at room temperature is ±0.5–3.5 °C. The error was calculated from 18 different readings with an average error of ±0.15 °C.
(c) The IoT network can be developed to be heterogeneous in the physical layer. However, with the IoT, the term heterogeneous also refers to the ability of the platform to be able to communicate with widely different devices. Here a heterogeneous IoT broker was developed via an embedded system without any needs to develop a mixed signal chip. The study shows that, the IoT broker can communicate with complex IoT nodes with different frequencies and with the cloud computing in real-time without latency. This communication algorithm was developed using sequential programming based on “interrupt”, without any need to use a real-time operating systems (RTOS).
(d) The design of software has a high impact on enhancing the data acquisition algorithm and minimizing the usage of the processing power of the hardware platform as well. The effective development of software makes the system able to deploy on low specification platforms such as embedded Linux kits and microcontrollers.
(e) The next generation e-healthcare systems is foreseen, especially in the light of recent development of metaverse, AI, VR, AR and smart non-invasive sensing techniques. The transition from the R&D mode into a commercial business model is envisioned and expected to pave the way for reliable, cost efficient, and rapid smart healthcare systems.

## Figures and Tables

**Table 1:** Different IoT technologies in vital data acquisition systems.

Technology	Merits	Demerits	Used in
LoRa	Low energy consumption, long-range operable standard	Low data size and volume	[30,39]
SigFox	Low energy consumption, long range, higher spectral efficiency, low noise	Supports one-way communication without acknowledgment, low data rate	[61]
Zwave	Low Interferences, power efficiency	Implementation cost of network, difficulty in configuration, performance issues with the number in nodes, limited number of nodes	[62]
RFID	No wave emission No need for energy	Low range	[63]
Bluetooth	Message size and volume debit	Low range	[64]
Wi-Fi	High data rate, secure communication	High power consumption, need for gateway	[30,39]
ZigBee	Secure connection, low power low cost, high range	Low data rate Short range	[65]

**Table 2:** Different sensing techniques and technologies for human vital signs.

Sensors	Interfacing	Features	Vital Signs	Measuring Methodology
MySignals (Commercial sensor) [156]	WiFi and BLE Integrated with Arduino and Raspberry Pi	Unwearable	Body Position, Body Temperature, Electromyography, Electrocardiography, Airflow, Galvanic Skin Response, Blood Pressure, Pulse Oximeter Glucometer, Spirometer, Snore Scale, Electroencephalography	Invasive
e-Health V2.0 (Commercial sensor) [156]	WiFi and BLE Integrated with Arduino and Raspberry Pi	Wearable as T-shirt	Patient Position Sensor (Accelerometer) Glucometer Sensor, Body Temperature Sensor, Blood Pressure Sensor (Sphygmomanometer) V2.0, Pulse and Oxygen in Blood Sensor (SPO2), Airflow Sensor (Breathing), Galvanic Skin Response Sensor (GSR-Sweating), Electrocardiogram Sensor (ECG), Electromyography Sensor (EMG)	Invasive
WEALTHY (Developed sensor) [157]	Analog-to-digital Converter (ADC) Can be interfacing with microcontroller	Wearable	Electrocardiogram, Respiration, Activity	Invasive
Wearable sweat sensors (Developed sensor) [158]	Analog-to-digital Converter (ADC) Can be interfacing with microcontroller	Wearable on hand above skin	Diabetes	Partially non-invasive
Thermal Camera (Developed algorithm) [147,148]	Computer Embedded Linux kit	Utilizing thermal camera	Body Temperature	Partially non-invasive
RGB Camera and IR Sensor (Developed algorithm) [30,39]	Computer Embedded Linux kit Microcontroller	Utilized thermal camera and Infrared sensor	Heart rate and Body Temperature	Non-invasive
3D Camera (Developed algorithm) [134]	Computer	3D imaging	Heart Rate and Oxygen Saturation	Non-invasive

**Table 3:** Different smart e-healthcare applications based on IoT and invasive/non-invasive data acquisition.

Work	Non-Invasive	IoT	Wireless Technology	Sensing Technology	Measured Vital Data	Advantages	Limitations
[49]	No	No	N/A	Piezoresistive	BP, Heart rate	High accuracy, short measurement time, portable	Very sensitive
[52]	No	Yes	GPS, GSM	Analog, optical	Heart rate, BT	Direct tracking of patients, SMS iseasier to access	Bulky circuitry, movement artifacts may affect accuracy of heart rate sensor
[113]	No	Yes	2.4 GHzradio	Infrared LED with phototransistor	Heart rate	Comfortable	Looseness leads to movement artifacts,
[118]	Yes	No	No	CW Doppler radar	Respiration rate, Heartrate	Non-contact measurement	Easy interference by noise
[120]	Yes	No	N/A	CW Doppler radar	Heart rate	High accuracy, non-contact measurement	Must be readjusted in different environments
[121]	No	No	No	Strain gauges, dry electrodes	BCG, ECG	Convenient, safe	Not portable, PC is required
[132]	Yes	No	N/A	Piezoelectric pulse transducer, middle-wavelength IRcamera	Cardiovascular pulse	Non-contact measurement, high accuracy	Environment may seriously affect the performance
[133]	No	No	N/A	IR thermal camera	Respiration rate, heart rate (in sheep)	Non-contact measurement	Animal use only
[134]	Yes	No	N/A	3D camera	Temperature, respiration rate, heart rate, SpO_2_	Non-contact measurement, real-time	Low accuracy
[63]	No	No	RFID	Digital sensor	Temperature	Battery-free, compact	Requires a reading device
[142]	No	Yes	Wi-Fi	Non-contact IR sensor	Temperature	Non-contact measurement, Robust Wi-Fi	Low reliability
[145]	No	No	2.4 GHz radio	Digital sensor	Temperature	High accuracy, low power, low cost	The difference in temperature is approximated
[64]	No	Yes	Bluetooth	Digital sensors	Temperature, humidity	Critical data are extracted from simple data	Indirect connection to internet
[148]	Partially	No	N/A	Thermal camera, wristband	Temperature	High accuracy	Low reliability with sweat requires uses of a reference device
[151]	No	No	N/A	Magnetic induction	Human activity recognition	Lightweight, portable, cheap, high accuracy	Cross-coupling may be destructive
[30,39]	Yes	Yes	LoRa and WiFi	RGB Camera	Heart Rate	Low Processing power, portable, safe, easy to use, high range of sending data	Very sensitive for environment light
[39]	Yes	Yes	LoRa and WiFi	IR Sensor	Body temperature	Portable, easy to use, high range of sending data, low cost	Needs short distance to be used
[159]	No	Yes	Bluetooth and mobile phone	Photoplethysmography	Body Temperature, oxygen saturation, heart rate, and respiratory	Enable remote monitoring Easy to be integrated with mobile phone	Needs to be worn
[160]	No	Yes	BT/BLE	Pulse oximetry sensor	oxygen saturation	Secured connection	Needs to be worn

**Table 4:** Business model for e-healthcare system based on IoT and non-invasive data acquisition.

Business Model Components	Description
Product Description	Sense and recognize vital sign of human using surveillance camera/mobile phone camera and visualize and analyze the acquired vital signs in cloud-based applications. Moreover, the facility provides medical consultation and investigating for remote patients using metaverse.
Customer needs	Real-time visualization and analysis of their vital signs on their mobile phone application without need to wear/touch sensors as well as the ability to meet doctors in metaverse and perform investigation.
Technologies	IoT, non-invasive data acquisition, cloud computing, combined AI and metaverse application
Human resources	embedded systems engineers, computer vision engineers, augmented reality engineers, pre-sales engineers, marketing employers, sales employers, technical support employers and customer services employers.
Financial	Looking for funding agency and partnership with health insurance companies.

## Data Availability

Not Applicable.
